# Infectious diseases may have arrested the southward advance of microblades in Upper Palaeolithic East Asia

**DOI:** 10.1098/rspb.2023.1262

**Published:** 2023-08-30

**Authors:** Kenichi Aoki, Naoyuki Takahata, Hiroki Oota, Joe Yuichiro Wakano, Marcus W. Feldman

**Affiliations:** ^1^ Graduate School of Science, University of Tokyo, Hongo, Tokyo 113-0033, Japan; ^2^ Graduate University for Advanced Studies, Hayama, Kanagawa 240-0116, Japan; ^3^ Department of Biological Sciences, Graduate School of Science, University of Tokyo, Hongo, Tokyo 113-0033, Japan; ^4^ School of Interdisciplinary Mathematical Sciences, Meiji University, Nakano, Tokyo 164-8525, Japan; ^5^ Department of Biology, Stanford University, Stanford, CA 94305, USA

**Keywords:** microblades, ancient DNA, diffusion equation, infectious disease, Qinling–Huaihe Line

## Abstract

An unsolved archaeological puzzle of the East Asian Upper Palaeolithic is why the southward expansion of an innovative lithic technology represented by microblades stalled at the Qinling–Huaihe Line. It has been suggested that the southward migration of foragers with microblades stopped there, which is consistent with ancient DNA studies showing that populations to the north and south of this line had differentiated genetically by 19 000 years ago. Many infectious pathogens are believed to have been associated with hominins since the Palaeolithic, and zoonotic pathogens in particular are prevalent at lower latitudes, which may have produced a disease barrier. We propose a mathematical model to argue that mortality due to infectious diseases may have arrested the wave-of-advance of the technologically advantaged foragers from the north.

## 1. Introduction

### (a) Microblades

Microliths are small crescentic, triangular or trapezoidal-shaped stone tools, with the bulging edge blunted. They first appear in the archaeological record at various Middle Stone Age sites in southern Africa, and, by analogy with ethnographical examples, are thought to have been used as tips and barbs of wooden projectiles [1,2], facilitating the capture of fast-moving prey [3]. Microblades, on the other hand, are small blades less than approximately 5 cm in length, detached from specially prepared cores [4]. They are not regarded as formal stone tools [5], but can yield microliths when retouched.

There is much controversy as to whether microlithic industries based on microblades or on slightly larger bladelets, found in distant regions of the world, have independent origins [2,6]. The geographical origin of the microblades that we refer to in this study, which dates to approximately 30–25 kya (kya is short for 1000 years ago) or perhaps approximately 40–35 kya [4], has been variously placed in the Amur River Basin [7], Siberia [8], the Mammoth Steppe [9], the Far East [4] or Northeast Asia [10]. These microblades are often characterized by their novel method of removal from the microblade core, namely by pressure-flaking rather than by percussion-knapping [4,11]. In what follows, we will simply refer to microblades of this description as microblades.

The question we address is why microblades spread as far south as the Qinling–Huaihe Line that separates northern and southern China (latitude 33°N), but no further, during the Upper Palaeolithic. Qu *et al*. [8 their figs 1 and 2] show the geographical distributions of dated microblade sites in East Asia in the time ranges approximately 35–23/22 kya and approximately 21/20–12/11 kya, respectively. A comparison of these two figures clearly shows a southward expansion of microblades, but no microblade assemblages occur in southern China and Southeast Asia into the early Holocene [12], although suitable lithic materials such as flint and chert were available [11,13]. Middle Holocene sites such as Zhongzipu in Sichuan Province [14] and Xiqiaoshan in Guangdong Province [15] have, however, yielded microblades (see also [16, table 2]), which may be attributable to increased migratory activity in the Neolithic [17,18].

Bar-Yosef *et al*. [12] review various hypotheses that have been proposed to explain this conundrum. Among them the ‘bamboo hypothesis’ may be the most famous, but as pointed out in the same paper, the manufacture and use of non-lithic (bamboo) tools—note bamboo is perishable and not detectable in the archaeological record—does not preclude the adoption of innovative stone technologies. The simple answer according to Bar-Yosef [11] is that ‘the people of the north never moved south.’ But why not? We hypothesize that mortality and/or infertility caused by infectious diseases that were widespread in the south may have been the reason.

Ammerman & Cavalli-Sforza [19–21] pioneered the application of wave-of-advance/reaction–diffusion models [22–26] to archaeological issues [27,28]. Their point of departure was the demonstration of an approximately constant rate at which early farming spread from the Near East throughout much of Europe. If we invoke the Fisher [29] model, this observation is consistent with a scenario in which the spread of early farming across Europe was mediated by the range expansion of farmers of Near Eastern origin. Ancient DNA studies have shown that early Neolithic individuals from as far west as Spain were in fact of Anatolian origin [30–35].

What is empirically perhaps more relevant from our standpoint is an ancient DNA study showing that genetically distinct groups of hunter–gatherers and farmers coexisted for a long time [36]. An archaeological study by Vanmontfort [37] also suggests that range expansion does not always occur, but that static boundaries between different populations may be maintained over a long time span. Such a boundary is known in reaction–diffusion theory as a ‘stationary front’.

In the Levant, the inter-species boundary between Neanderthals and modern humans remained geographically localized for tens of thousands of years during the Middle Palaeolithic—although not entirely impervious (approx. 54 kya Mandrin in France, [38]). Greenbaum *et al*. [39] invoke infectious diseases specific to Neanderthals and modern humans—caused by Eurasian and African pathogens, respectively—to account for the mutual inability to extend their ranges. They propose epidemic models that incorporate adaptive genetic introgression and argue that modern humans may have been able to overcome their disease load before the Neanderthals and hence commence an enduring out-of-Africa dispersal.

In the Upper Palaeolithic of East Asia, a population equipped with microlithic technology (based on pressure-flaked microblades) may have had a demographic edge over a population solely dependent on core-and-flake or cobble tools (and perhaps bamboo implements). This demographic edge is likely not to have been as pronounced as that of the early farmers over hunter–gatherers, which may have entailed a greater than 100-fold increase in carrying capacity [21,40]. Mellars & French [41] estimate a 10-fold population increase during the Mousterian to Aurignacian transition in southern France, partly attributable to technological advance. The southward expansion of microblades is consistent with Lotka–Volterra models of competition [26] between foragers with and without microblades; and it is difficult to explain why this southward expansion was suspended without assuming some critical difference between the environments of, and/or the populations inhabiting, northern and southern East Asia.

In this paper, we first review the recent genetic literature on the peopling of East Asia by modern humans that began approximately 45 kya. We emphasize that a north–south genetic differentiation may have been established, at least among the ‘coastal’ populations, by approximately 19 kya at the latest. Second, we review the evidence for parasitic and infectious diseases that may have afflicted hominins since the Palaeolithic. We focus on diseases that may have had their origins in southern East Asia or Southeast Asia, noting that the majority of zoonotic diseases are currently found in low latitude regions of the world. Third, we propose and analyse a wave-of-advance/reaction–diffusion model that incorporates a simplified version of the single-timescale non-spatial epidemiological dynamics assumed by Greenbaum *et al*. [39]. By adding the diffusion (i.e. migration) term, it is explicitly shown that a stationary front may be formed between the populations of northern and southern East Asia.

### (b) Genetic aspects of the peopling of East Asia

A single major migration of modern humans into the continents of Asia and Sahul was strongly supported by earlier studies using mitochondrial DNA, the non-recombining portion of Y chromosomes, and autosomal SNP data [42–45]. Ancestral Ancient South Indians with no West Eurasian relatedness, East Asians, Onge (Andamanese hunter–gatherers) and Papuans all derive in a short evolutionary time from the eastward dispersal of an out-of-Africa population [46,47], although Europeans and East Asians are suggested to share more recent common ancestors than with Papuans [48]; but see [49].

The HUGO (Human Genome Organization) Pan-Asian SNP consortium [44] investigated haplotype diversity within present-day Asian populations and found a strong correlation with latitude, with diversity decreasing from south to north. The correlation continues to hold when only mainland Southeast Asian and East Asian populations are considered, and is perhaps attributable to a serial founder effect [50]. These observations are consistent with the view that soon after the single eastward migration of modern humans, East Asians diverged in southern East Asia and dispersed northward across the continent.

A key ancestry in Upper Palaeolithic East Asia is represented by the approximately 40 kya Tianyuan (TY) individual excavated outside Beijing [51,52], together with the closely related approximately 34 kya Salkhit individual from eastern Mongolia [53], and the approximately 33 kya AR33K individual from the Amur River Basin [54]. By 40–33 kya, northern China and Mongolia were inhabited by populations with TY ancestry that were strongly differentiated from the populations of Southeast Asia. The TY ancestry became widespread geographically before the Last Glacial Maximum (LGM approx. 26.5–19 kya), but most TY-related populations were soon replaced by populations such as the one represented by the approximately 19 kya AR19K individual [54].

The TY individual was recently found to have belonged to a sister group of the approximately 45 kya Bacho Kiro individuals in Bulgaria, and its genetic affinity to approximately 35 kya Goyet Q116-1 in Belgium is explained by shared ancestry [55,56]. This shared ancestry of East Eurasian populations before approximately 45 kya implies that admixture in northern East Asia between those who separately took the northern and southern routes relative to the Himalayas, if indeed it occurred, cannot be easily detected because all individuals belonged to a genetically similar group. In fact, such admixture in northern East Asia is genetically demonstrated only after West Eurasian populations represented by Ancient North Siberians (ANS) and/or Ancient North Eurasians (ANE) migrated to northeast Siberia in the Upper Palaeolithic after approximately 40 kya [57–59].

Mao *et al*. [54] argue that populations in the Amur River Basin, i.e. coastal Northern East Asians (cNEA), played an important role in interactions with populations associated with ANS ancestry, although there is no evidence for admixture in the AR33K individual. More importantly in the present context, Mao *et al*. [54] suggest that ancient cNEA and ancient coastal Southern East Asians (cSEA), bounded by the Qinling–Huaihe Line, could already have been separated into two distinct genetic lineages by approximately 19 kya. This is because in their *qpGraph* and maximum-likelihood tree [54 their figs 3*a* and S3, respectively], the approximately 19 kya AR19K individual from the Amur River Basin, the earliest cNEA yet identified, is placed as a tip within cNEA groups after their earlier split from cSEA groups, represented in the former figure by approximately 7.5 kya Liangdao2. Huang *et al*. [60] estimate this divergence time to be 23.4 kya (i.e. earlier than 19 kya) and affirm the deep north–south split in East Asia [17,18].

With regard to cSEA, Yang *et al*. [17] find that Early Neolithic southern East Asians (e.g. Liangdao2 and approx. 11.5 kya Qihe3 in Fujian Province) are genetically distinct from contemporary northern East Asians (e.g. approx. 9.5 kya Bianbian in Shandong Province, approx. 8.4 kya Yumin in Inner Mongolia, Devil's Gate, and AR19K). Furthermore, Wang *et al*. [61] and Zhang *et al*. [62] show that apart from the Hòabìnhians (Southeast Asian hunter–gatherers), there were at least two more distinct ancestries in southern China represented by approximately 10.5 kya Longlin1, approximately 8.7 kya Dushan4 and approximately 7.4 kya Baojianshan5 in Guangxi Province, and by approximately 14 kya Mengzi Ren (MZR) in Yunnan Province. These southern East Asians form a clade that is clearly distinct from the northern clade comprising the Early Neolithic northern East Asians.

It is not known, and may be difficult to infer exactly, when the cNEA and cSEA lineages differentiated from each other, and similarly for the coastal and interior TY-related lineages. East Asian populations in the early Holocene are highly differentiated (fixation index *F*_ST_ = 0.067) compared to the present-day (*F*_ST_ = 0.013) [18]. Jomon hunter–gatherers in the Japanese archipelago may provide an upper bound on the split time between cNEA and cSEA. Jomon are basal to present-day East Asians and considered to be an out-group of ancient cNEA and ancient cSEA [17,54,63–65]. Kanzawa-Kiriyama *et al*. [66] infer that the split time between the Funadomari Jomon (3.8 − 3.5 kya) individual and Han (two individuals) ranges between 38 kya and 18 kya.

All available ancient East Asian genomes thus support the existence of some degree of genetic divergence between East Asian foragers north and south of the Qinling–Huaihe Line during or even before the LGM.

### (c) Parasitic and infectious diseases in Upper Palaeolithic East Asia

Various parasitic and infectious diseases, e.g. body lice [67–69], tapeworms [70,71], tuberculosis [72–74], malaria [75–80], papillomaviruses [81–83] and coronaviruses [84–87] are believed to have been associated with hominins since the Palaeolithic. Some of these diseases may have infected southern East Asian foragers, resulting in some population-level immunity and/or behavioural accommodation, whereas northern East Asian foragers would have been minimally exposed. By southern and northern foragers, we mean modern humans who, arriving by the southern route, populated East Asia as far north as TY, Salkhit and the Amur River Basin, then differentiated genetically into southern and northern populations by approximately 19 kya at the latest, with the approximate boundary at the Qinling–Huaihe Line [8,17,54] (see above). We provide a short account below of malaria and papillomaviruses because of their possible relevance, and of coronaviruses because of their current interest.

*Plasmodium vivax* malaria is not as lethal as *P. falciparum* malaria, but nevertheless highly debilitating (e.g. [88,89]) (see below). It was endemic throughout modern China, including the northern (above 33° N) and northwestern regions, until its recent eradication [90]. Based on a phylogenetic analysis of 10 *Plasmodium* species that have primates from various regions of the world as their natural hosts, Escalante *et al*. [77] infer a Southeast Asian origin for *P. vivax*. Moreover, they estimate the time to the most recent common ancestor (TMRCA) of extant *P. vivax* to be approximately 82–46 kya, a timeframe that may suggest an initial host switch from Asian macaques to archaic (and/or super-archaic) humans. An African origin of *P. vivax* has also been proposed [79] and, although controversial [80], we include *P. vivax* malaria among the diseases that may have infected modern humans in southern East Asia and/or Southeast Asia. Duffy allele FY*A, which is currently found at high frequency in East Asia, may confer some resistance to *P. vivax* malaria [80,91].

In a population-based study conducted in Papua, Indonesia over a 4 year period in the first decade of this century, Tjitra *et al*. [88] estimate the risk of death as 1 per 3959 diagnosed cases (0.025%) of *P. vivax* infection. By comparison, mortality from *P. falciparum* infection in the same study is 1 per 1742 (0.057%), which is about twice as high. They caution that both estimates may be conservative. A crude estimate of current *P. vivax* mortality (number of patients who died divided by the total number of patients aggregated over 75 articles) is 334/814 505 = 0.04%, whereas among hospitalized patients, mortality is an order of magnitude higher [89]. Moreover, malarial infection by either *P. vivax* or *P. falciparum* in pregnant women results in more miscarriages, stillbirths and neonatal mortality [89]. In the Palaeolithic, the debilitating effects of malarial infection such as high fever would have imposed a burden on foraging activities and possibly contributed to death by starvation.

**Figure 1.**
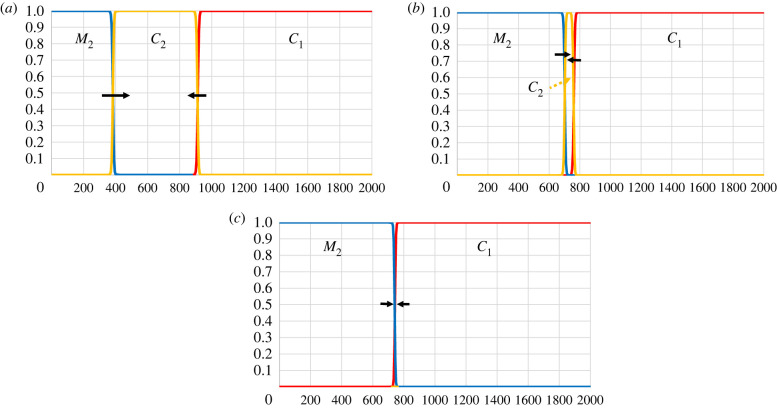
Numerical solution of the non-dimensional equations (2.4, 2.5) by the forward Euler method with Neumann boundary conditions on a space lattice comprising l+1 points where l=2000; space and time increments are Δx=0.5 and Δt=0.05, respectively. Initial conditions are $C_{1}(x,0)=1$ for l/2+1≤x≤l and 0 elsewhere; $M_{2}(x,0)=1$ for 0≤x≤l/10 and 0 elsewhere; $C_{2}(x,0)=1$ for l/10+1≤x≤l/2 and 0 elsewhere. Parameter values are $L_{1}=50$, $K_{2}=100$, $L_{2}=60$, b=0.825, ρ=1.2, ε=0.01, w=0.3, δ=0.029053. Densities of $C_{1}$, $M_{2}$ and $C_{2}$ are pictured in red, blue and yellow, respectively. (*a*) Solution after 1500 time steps (iterations); (*b*) after 4000 time steps; (*c*) after 4500 time steps. With $r_{1}=0.025$ (<0.03 to reflect disease load), one time step corresponds to 2 years. At about 4500 time steps, i.e. 9000 years, a stationary front has been formed at x≈743 between $M_{2}$ on the left (north) and $C_{1}$ on the right (south). The simulation was continued until 6000 time steps as a check.

*Human papillomavirus* 16 (HPV16) is an oncogenic infectious agent, in particular causing cervical cancer. Pimenoff *et al*. [82] effectively distinguish five HPV16 lineages, A1–A3, A4, B, C and D; among these A4 is the most common lineage in East Asia and virtually absent elsewhere. They then show that the A1–A3 and A4 lineages both diverged early from the sister B, C, D lineages (*ca* 600 − 400 kya), and that A1–A3 and A4 have a more recent TMRCA and encompass less genetic diversity than the B, C and D lineages [82, fig. 1]. Based on these observations, they argue that a host switch of the A1–A3 and A4 lineages by sexual transmission took place from archaic humans inhabiting Eurasia to out-of-Africa modern humans.

Chen *et al*. [83] invoke analogous observations on HPV58 to explain their phylogeography. They suggest that the A1 and A3 sublineages of HPV58 may have become predominant in East Asians due to admixture with Oceanians who were previously infected by Denisovans. Given the complexities of modern human ancestries [92], an alternative scenario of the codivergence of *human papillomaviruses* 16 and 58 with out-of-Africa modern humans cannot be ruled out, but it is quite possible that infection of modern humans by HPV16A4, HPV58A1 and HPV58A3 occurred in southern East Asia and/or Southeast Asia, possibly from Denisovans.

HPV prevalence among men usually exceeds 20% [93]. Among women in China, prevalence was 26% in the second decade of this century, and the second and third most common types were HPV16 and 58 (see above), respectively [94]. In 2020 in China, the incidence of cervical cancer was 10.7 per 10^6^ women-years and mortality was 5.3 per 10^6^ women-years, respectively, and the corresponding rates for Southeast Asia were 17.8 and 10.0, respectively [95, table]. From the same table, a crude estimate of mortality in cervical cancer cases is 59 060/109 741 = 54% in Chinese women and 38 530/68 623 = 56% in Southeast Asian women. Mortality due to cervical cancer ranges from 3.1 to 19.8 per 10^6^ women-years among regions with high and low Human Development Index levels [95, table], and would most likely have been appreciably higher in Palaeolithic societies when medical intervention was unavailable. In addition, HPV infection in either the female or male partner may adversely affect the initiation and outcome of pregnancies [96,97].

An interesting recent finding is that modern humans in East Asia may have been infected with coronavirus at least approximately 20 kya [84,87]. The evidence comprises signatures of selection on genes that code for coronavirus-interacting human proteins (CoV-VIPs). Among the 26 populations from the 1000 Genomes Project dataset (1000 Genomes Project [98]), Souilmi *et al*. [84] observe selective sweep signals for multiple CoV-VIPs in the five East Asian populations, but none of the other populations. Souilmi *et al*. [84] estimate that the start of selection on the 42 CoV-VIPs clusters to around 970–770 generations ago, with the peak signature at 870 generations ago. That is, given a hunter–gatherer generation time of 29 years [99,100], selection may have started approximately 870 × 29 = approximately 25 kya. The implication is that all East Asians would have acquired some degree of immunity to coronavirus infection. Hence, adaptation to coronaviruses may have occurred concurrently in already diverging southern and northern East Asian populations.

Upper Palaeolithic East Asians were likely afflicted with one or more of these infectious diseases, as well as others not mentioned or yet to be discovered, and the combined disease load may have been substantial. Given the available evidence, in particular the uncertainties associated with date estimation, it is difficult to identify those diseases that became prevalent only among the southern foragers *after* their divergence from the northern foragers. We speculate that *Plasmodium vivax* malaria and *Papillomavirus* infection may be candidates for such diseases. The latitudinal bias in the distribution of zoonotic pathogens strongly suggests that ancient southern East Asians and Southeast Asians would have experienced a heavier disease load than northern East Asians [101–103], which could have produced an infectious disease barrier that prevented further southern expansion by lineages from the north. Although the recent dengue epidemics are not within the timeframe of our study, the characterization of this disease as mosquito-borne tropical/subtropical with a possible origin in Southeast Asia (e.g. [104,105]) suggests that it has the properties of the kind of disease discussed here.

## 2. Results

### (a) Minimal model

We first illustrate our approach with a highly-simplified model, which we will call the minimal model. All southern foragers are assumed to carry a disease (or diseases) to which they have tolerance (possibly asymptomatic); disease load among southern foragers may however be reflected in low relative intrinsic growth rate and/or carrying capacity. All northern foragers are susceptible and infection is immediately lethal; northern foragers who die of the disease(s) do so before producing secondary infections, and all surviving northern foragers are healthy.

Let $N_{1}(x,t)$ and $N_{2}(x,t)$ be the densities of southern foragers and northern foragers, respectively, at location *x* in a north–south one-dimensional space at time *t*. The reaction–diffusion equations of the minimal model are

2.1*a* $$\frac{\partial N_{1}}{\partial t}=D\frac{\partial^{2}N_{1}}{\partial x^{2}}+r_{1}N_{1}\left[1-\frac{N_{1}+b_{12}N_{2}}{L_{1}}\right]$$

and

2.1*b* $$\frac{\partial N_{2}}{\partial t}=D\frac{\partial^{2}N_{2}}{\partial x^{2}}+r_{2}N_{2}\left[1-\frac{N_{2}+b_{21}N_{1}}{K_{2}}\right]-mN_{1}N_{2}.$$

The left-hand side in each of equations (2.1*a*) and (2.1*b*) represents the rate of change with time of the respective densities at location *x*, and is equal to the sum of two terms on the right-hand side in the case of equation (2.1*a*) and of three terms for equations (2.1*b*). The first term on the right-hand side gives the effect of diffusion (random bidirectional migration; *D* is the diffusion coefficient). The second term represents logistic growth with Lotka–Volterra competition. Here, $r_{1}$ and $r_{2}$ are the respective intrinsic rates of growth; $L_{1}$ and $K_{2}$ the respective carrying capacities; $b_{12}$ and $b_{21}$ the competition coefficients. (Here and in the three-population model described below, we use subscripts 1 and 2 to denote southern and northern foragers, respectively, which reflects the order in which East Asia was populated by Upper Palaeolithic humans; we use letters *K* and *L* to distinguish the carrying capacities of forager groups with and without microblades.)

Parameter *m* is the contagion-mortality rate of the disease. More precisely, *m* is the fraction of susceptibles (northern foragers) that are infected—and as a result die—per unit time by one infected individual (southern forager). Note our assumption eliminates the infected-but-live stage in the classical Kermack–McKendrick model [106] among northern foragers. The rate of contagion, whether by direct body contact or by intermediaries such as mosquitoes, is assumed to be proportional to the product of the densities of infected and susceptible persons in the same neighbourhood ($N_{1}N_{2}$). Equations (2.1*a,b*) are in fact Lotka–Volterra competition–diffusion equations [26], but are written in this form to emphasize the additional mortality among northern foragers due to infection by southern foragers. Infertility among northern forager females and males caused by HPV infection, for example, would have had an analogous effect to mortality in hindering the wave-of-advance of the northern foragers. In addition, lowered fertility may perhaps have reduced gene flow. All parameters are assumed positive, with $\;b_{12}<1$ and $b_{21}<1$.

In electronic supplementary material, S1, we rewrite equations (2.1) in non-dimensional form and analyse the non-spatial version (i.e. without the diffusion term) of these equations. We determine the local stability properties of the four equilibria and show that, if $b_{12}K_{2}/L_{1}>1$ and $r_{2}(1-b_{21}L_{1}/K_{2})<mL_{1}$, the two corner equilibria, ${\hat{N}}_{1}=L_{1}$, ${\hat{N}}_{2}=0$ and ${\hat{N}}_{1}=0$, ${\hat{N}}_{2}=K_{2}$, (E-1 and E-2, respectively, in the notation of electronic supplementary material, S1) are both locally stable. The above assumption that disease load may lower carrying capacity entails that $b_{12}K_{2}/L_{1}>1$ if $L_{1}$ is sufficiently small relative to $K_{2}$. This suggests that a bistable travelling wave can exist in the spatial model (equations (2.1)), with northern foragers ($N_{2}$) dominant in the north (small *x*) and southern foragers ($N_{1}$) dominant in the south (large *x*). In particular, parameter values may exist for which the speed of the wave of advance is zero, i.e. the front is stationary.

### (b) A three-population model

Here we describe a more-detailed model that distinguishes foragers with and without microblades and allows for limited cultural conversion. We consider only the non-spatial dynamics here. Numerical analysis of the spatial model will be presented later.

Let $M_{1}(t)$ and $C_{1}(t)$ be the densities of southern foragers with and without microblades, respectively, at time *t*. Similarly, let $M_{2}(t)$ and $C_{2}(t)$ be the densities of northern foragers with and without microblades. Foragers without microblades have only core-and-flake or cobble tools (and possibly bamboo implements; the symbols *M* and *C* are mnemonic for microblades and core-and-flake/cobble tools, respectively). We do not incorporate cultural transmission from northern to southern foragers, which would produce a disagreement with the observed patterns of microblade appearance. Archaeological evidence suggests that initially only northern foragers had microblades, i.e. that $M_{1}(0)=0$ and $M_{2}(0)>0$. Hence, $M_{1}(t)=0$ for all t≥0, which yields a three-population model [107,108] with variables $C_{1}(t)$, $M_{2}(t)$ and $C_{2}(t)$. The nature and consequences of this restrictive assumption require further consideration.

We model cultural conversion among northern foragers by the density-dependent term

2.2*a* $$eM_{2}C_{2}P,$$

where

2.2*b* $$P=\frac{M_{2}}{C_{1}+M_{2}+C_{2}}-w,$$

and e>0. The newly introduced parameter w (0<w<1) represents a conformist effect, such that northern foragers with only core-and-flake or cobble tools adopt microblades when *P* is positive, while northern foragers abandon microblades when *P* is negative.

As before, we assume that all southern foragers bear a disease load, whereas all northern foragers are susceptible. With these assumptions, we write the non-spatial dynamics as

2.3*a* $${\dot{C}}_{1}=r_{C1}C_{1}\left[1-\frac{C_{1}+b_{12M}M_{2}+b_{12C}C_{2}}{L_{1}}\right]\;,$$

2.3*b* $${\dot{M}}_{2}=r_{M2}M_{2}\left[1-\frac{b_{21C}C_{1}+M_{2}+b_{22C}C_{2}}{K_{2}}\right]+eM_{2}C_{2}P-mM_{2}C_{1}$$

2.3*c* $$\mathrm{and}\;{\dot{C}}_{2}=r_{C2}C_{2}\left[1-\frac{b_{21C}C_{1}+b_{22M}M_{2}+C_{2}}{L_{2}}\right]\;-eM_{2}C_{2}P-mC_{2}C_{1}.$$

The parameters $r_{C1}$, $b_{12M}$, $L_{1}$, *m* etc. are analogous to those in equations (2.1). Three non-extinction corner equilibria, E-${\hat{C}}_{1}\;({\hat{C}}_{1}=L_{1},{\hat{M}}_{2}={\hat{C}}_{2}=0)$, E-${\hat{M}}_{2}\;({\hat{C}}_{1}=0,{\hat{M}}_{2}=K_{2},{\hat{C}}_{2}=0)$ and E-${\hat{C}}_{2}\;({\hat{C}}_{1}={\hat{M}}_{2}=0,\;{\hat{C}}_{2}=L_{2})$, always exist. The Jacobian matrix of equations (2.2), (2.3) is given in electronic supplementary material, S2, together with the necessary and sufficient conditions for local stability of these three equilibria.

### (c) Numerical analysis of the three-population model with spatial structure

The three variables, $C_{1}$, $M_{2}$, $C_{2}$, and hence also *P*, are now functions of both space, *x*, and time, *t*. We reduce the number of parameters to facilitate numerical analysis. Set $r_{C1}=r_{1}$, $r_{M2}=r_{C2}=r_{2}$, and all competition coefficients, e.g. $b_{12M}$, equal to *b*. We write the reaction–diffusion equations in terms of the transformed variables $t^{*}=r_{1}t$, $x^{*}=x\sqrt{r_{1}/D}$, $C_{1}^{*}=C_{1}/L_{1}$, $M_{2}^{*}=M_{2}/K_{2}$, $C_{2}^{*}=C_{2}/L_{2}$.

Then, suppressing the asterisks and noting that *P* becomes

2.4 $$P=\frac{K_{2}M_{2}}{L_{1}C_{1}+K_{2}M_{2}+L_{2}C_{2}}-w,$$

we obtain

2.5*a* $$\frac{\partial C_{1}}{\partial t}=\frac{\partial^{2}C_{1}}{\partial x^{2}}+C_{1}\left[1-C_{1}-\frac{b(K_{2}M_{2}+L_{2}C_{2})}{L_{1}}\right],$$

2.5*b* $$\begin{array}{rl} \frac{\partial M_{2}}{\partial t}= & \frac{\partial^{2}M_{2}}{\partial x^{2}}+\rho M_{2}\left[1-M_{2}-\frac{b(L_{1}C_{1}+L_{2}C_{2})}{K_{2}}\right]\\ & +\varepsilon L_{2}M_{2}C_{2}P-\delta M_{2}L_{1}C_{1} \end{array}$$

2.5c $$\begin{array}{rl} \mathrm{and}\;\frac{\partial C_{2}}{\partial t}= & \frac{\partial^{2}C_{2}}{\partial x^{2}}+\rho C_{2}\left[1-C_{2}-\frac{b(L_{1}C_{1}+K_{2}M_{2})}{L_{2}}\right],\\ & -\varepsilon K_{2}M_{2}C_{2}P-\delta C_{2}L_{1}C_{1} \end{array}$$

where $\rho =r_{2}/r_{1}$, $\varepsilon =e/r_{1}$ and $\delta =m/r_{1}$. In choosing relevant values for the transformed parameters in equations (2.4) and (2.5) it is useful to note that the intrinsic growth rate in modern humans is approximately 3% per year (e.g. [21]) and that local group sizes of ethnographic foragers range from perhaps 15–250 [109–112]. In the simulations of equations (2.4), (2.5), we assume (see above) that the intrinsic growth rates of $M_{2}$ and $C_{2}$ are equal $\left(r_{M2}=r_{C2}\right)$, which allows them to grow at the same rate when they are rare. However, the carrying capacity of $M_{2}$ is set larger ($K_{2}>L_{2}$), so competition favors $M_{2}$ over $C_{2}$, which reflects our postulated advantage of microblades.

### (d) A numerical example consistent with archaeological observations

**Figure 2.**
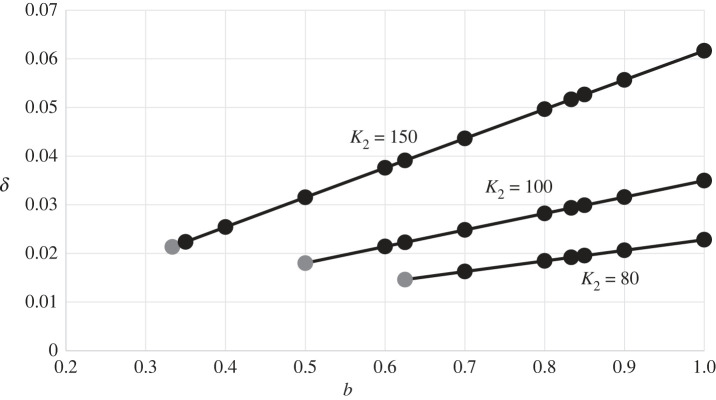
Values of the contagion-mortality parameter, $\delta =m/r_{1}$, that yield a stationary front between $M_{2}$ and $C_{1}$ are apparently linearly increasing in the competition coefficient, *b*, for each of three values of $K_{2}$. Fixed parameters are $L_{1}=50$, $L_{2}=60$, ρ=1.2, ε=0.01, w=0.3. The grey dot corresponds to the case where $1-(b/K_{2}+\delta /\rho )L_{1}=0$ and $1-bK_{2}/L_{1}=0$. Electronic supplementary material, equation S4a does not apply when $b>L_{1}/L_{2}=5/6$. Results were obtained by numerical solution of equations (2.4, 2.5) rather than of equations (2.6), from the same initial conditions as in figure 1, to ensure that $M_{2}$ or $C_{1}$ would not be eliminated.

Archaeological evidence (e.g. [8, fig. 1] suggests that as a first approximation we may adopt the following initial conditions (in terms of the transformed variables): $M_{2}=1$ and $C_{1}=C_{2}=0$ at the extreme northern (left) end of the linear space; $C_{1}=1$ and $M_{2}=C_{2}=0$ in roughly the southern (right) half; $C_{2}=1$ and $C_{1}=M_{2}=0$ in between. From these initial conditions, two temporary wave fronts will be formed; the first, as viewed from the south (right), between $C_{2}$ and $C_{1}$, and the second between $M_{2}$ and $C_{2}$. Genetic evidence [17,54] suggests that the first front moved little during the post-LGM Palaeolithic (between approx. 20 kya and approx. 10 kya). On the other hand, the archaeological evidence [8, figs 1 and 2] suggests that the second front moved south, and that $M_{2}$ had displaced $C_{2}$ before the Holocene (by approx. 11 kya at the latest), eventually resulting in the formation of a stationary front between $M_{2}$ and $C_{1}$. Numerical analysis of our model predicts such behaviour.

Figure 1 illustrates one numerical solution as it develops through time. The figure reveals three typical properties of archaeologically relevant solutions that may be obtained depending on the choice of parameter values. First, $C_{1}$ (southern foragers without microblades) will spread north (left) displacing $C_{2}$. Second, $M_{2}$ (northern foragers with microblades) will spread south (right) displacing $C_{2}$ (northern foragers without microblades). Third, $C_{2}$ eventually disappears and a stationary front is apparently formed where $C_{1}$ and $M_{2}$ collide. The values assigned to the carrying capacities, $L_{1}$, $K_{2}$, and $L_{2}$, here and below reflect the composition of what is known about present-day foragers [109–112]. The speeds of the two waves of advance—$C_{1}$ and $M_{2}$ each displacing $C_{2}$—can be predicted under certain conditions (electronic supplementary material, S3). As a consequence of our simplifying assumption that infections are immediately lethal, deaths (and/or infertility) among northern foragers due to these infections are predicted to occur only within a narrow zone of contact/coexistence with southern foragers (figure 1).

### (e) Parameter values that yield a stationary front

Our objective here is to numerically estimate combinations of parameter values that yield a stationary front between $C_{1}$ and $M_{2}$ in the absence of $C_{2}$. Setting $C_{2}=0$ in equations (2.5*a*) and (2.5*b*) and rewriting the latter in Lotka–Volterra form, we obtain

2.6*a* $$\frac{\partial C_{1}}{\partial t}=\frac{\partial^{2}C_{1}}{\partial x^{2}}+C_{1}\left[1-C_{1}-\left(\frac{bK_{2}}{L_{1}}\right)M_{2}\right]$$

and

2.6*b* $$\frac{\partial M_{2}}{\partial t}=\frac{\partial^{2}M_{2}}{\partial x^{2}}+\rho M_{2}\left[1-M_{2}-\left(\frac{b}{K_{2}}+\frac{\delta }{\rho }\right)L_{1}C_{1}\right].$$

Equations (2.6) are equivalent to equations (2.1) when the appropriate correspondences are made. We consider only the case where the two corner equilibria, E-${\hat{C}}_{1}$ and E-${\hat{M}}_{2}$ (see above), are both locally stable, which in terms of the parameters of the non-dimensional three-population model requires that $1\leq (b/K_{2}+\delta /\rho )L_{1}$ and $1\leq bK_{2}/L_{1}$. (These conditions correspond to the first sufficient condition for local stability of E-$C_{1}$ and E-$M_{2}$, respectively, in electronic supplementary material, S2.) Parameters $L_{2}$, ε and *w* are thus irrelevant, although electronic supplementary material, Eqs. S4a and S4b indicate that these parameters can affect the position of the stationary front.

Figure 2 apparently shows that a positive linear relation holds between the non-dimensional contagion-mortality, $\delta =m/r_{1}$, and the competition coefficient, *b*, for various values of the carrying capacity of northern foragers with microblades, $K_{2}$. If we assume an intrinsic growth rate of $r_{1}=0.025$ per year for the southern foragers, then a contagion-mortality rate of say $m=\;r_{1}\delta =0.025\times 0.04$ among northern foragers, which equals 1 per 1000 per year, may maintain a stationary front.

**Figure 3.**
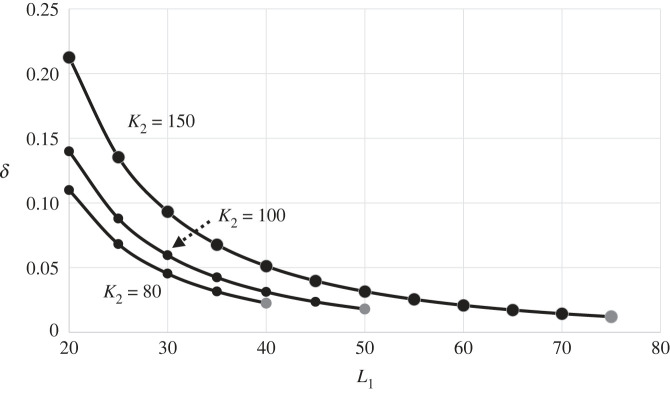
Values of the contagion-mortality parameter, $\delta =m/r_{1}$, that yield a stationary front between $M_{2}$ and $C_{1}$ are apparently inversely proportional to the square of the carrying capacity of southern foragers without microblades, $L_{1}$, for each of three values of $K_{2}$. Fixed parameters are $L_{2}=60$, b=0.5, ρ=1.2, ε=0.01, w=0.3. The grey dot corresponds to the case where $1-(b/K_{2}+\delta /\rho )L_{1}=0$ and $1-bK_{2}/L_{1}=0$. Results were obtained by numerical solution of equations (2.5) rather than of equations (2.6), from the same initial conditions as in figure 1, to ensure that $M_{2}$ or $C_{1}$ would not be eliminated.

Figure 3 shows how existence of a stationary front depends on parameters $L_{1}$, $K_{2}$ and $\delta =m/r_{1}$. Recall that parameter $L_{1}$ is the carrying capacity of southern foragers without microblades and is assumed to be relatively small. For each $K_{2}$, we find that $\delta =m/r_{1}$ is apparently quadratic in $1/L_{1}$. When $L_{1}$ is small relative to $K_{2}$, a high mortality among the northern foragers is required to prevent their further southward expansion and thus to maintain the stationary front.

### (f) Mathematical note

Kan-on [113] proved the existence and uniqueness of travelling wave solutions of equations (2.6), and some mathematical properties regarding the speed of these solutions. Applying these results to our model, a travelling wave solution of speed zero exists in the symmetric case where the intrinsic growth rates and competition coefficients of $C_{1}$ and $M_{2}$ are both identical, i.e. when ρ=1 and $(bK_{2}/L_{1})=(b/K_{2}+\delta /\rho )L_{1}$, respectively. Solving the latter equality for δ yields

2.7 $$\delta =\rho b\left(\frac{K_{2}}{L_{1}^{2}}-\frac{1}{K_{2}}\right).$$

We conjecture that equation (2.7) remains an approximate criterion for the existence of a stationary front for small deviations of ρ from 1 (electronic supplementary material, S4). Equation (2.7) entails that the disease-related mortality of northern foragers $\left(m=\;r_{1}\delta \right)$ is proportional to their intrinsic growth rate $\left(r_{2}=r_{1}\rho \right)$. Figures 2 and 3 are drawn for ρ=1.2. The numerical estimates of δ are, as noted above, apparently linear in *b* and quadratic in $1/L_{1}$ in agreement with equations (2.7). Extensive numerical work supports this conjecture, although the numerical estimate of δ is slightly smaller or larger than predicted by equation (2.7) when ρ>1 or ρ<1, respectively.

## 3. Discussion

Microblades are an advanced lithic technology invented somewhere in northernmost East Asia or perhaps Siberia [4,114]. They spread southward as evidenced by their discovery at various archaeological sites, and between approximately 21 and 11 kya had reached their southern limit defined by the Qinling–Huaihe Line [8]; the foragers to the south of the Qinling–Huaihe Line continued to rely on primitive core-and-flake and cobble tools, possibly supplemented by bamboo implements, into the early Holocene [12]. There is no doubt that the foragers in southern East Asia and Southeast Asia were modern humans, and lithic raw materials suitable for the manufacture of microblades were available there [12,13]. Coincidentally, (some of) the foragers to the north and south of the Qinling–Huaihe Line had differentiated genetically by approximately 19 kya at the latest [54]. Genetic differentiation of the northern and southern populations of East Asia was maintained until the Neolithic, when it was substantially reduced by increased migratory activity [17].

What stopped the southward expansion of the northern foragers with microblades? We propose and investigate the hypothesis that mortality (and/or infertility) due to infectious diseases prevalent in the south was the cause. After reviewing the literature on the genetic aspects of the peopling of East Asia by modern humans and the temporal–spatial distributions of several infectious diseases during the Palaeolithic, we formulate simple mathematical models that make explicit the structure of our hypothesis. Specifically, we use an approach based on wave-of-advance/reaction–diffusion models [22–26], which permits us to numerically obtain the conditions for the existence of a static disease barrier.

The models suggest that, if each southern forager were to have infected and caused the death of one in a thousand northern foragers per year, then the disease barrier could have been effective in arresting the southward spread of microblades. The total death rate of the northern foragers from the infectious disease(s) would have depended on the frequency of encounters with the southern foragers. Figure 1 suggests that such encounters would have occurred only at the interface between the spatial distributions of the southern and northern foragers. Hence, the total infectious-disease-related death rate among northern foragers may have been fairly low. On the other hand, our model neglects secondary infections among the northern foragers. The contagion-death rate required to sustain the barrier would also have been contingent on the carrying capacities, competition coefficients, etc., as illustrated in figures 2 and 3.

The pernicious effects of *Plasmodium vivax* [88,89] and *papillomavirus* infection [93–97] were briefly reviewed above. Both pathogens are known causes of mortality and pregnancy complications in humans and were likely present in East and Southeast Asia during the Upper Palaeolithic [76,77,82,83]. As described above, mortality estimates—the fraction of *P. vivax* malaria patients who die, and the death rate from cervical cancer in the total population—are not excessively high in modern populations. However, we suggest that without medical intervention both incidence and mortality rates in the Palaeolithic would likely have been higher. In addition, high fever associated with *P. vivax* malaria may have undermined foragers' ability to obtain food, resulting in death by starvation. The combined disease load from *P. vivax*, *papillomavirus*, and possibly other pathogens would most likely have had a significant demographic impact.

The role played by infectious diseases in shaping human history [115] and likely also their prehistory cannot be underestimated. Genetic studies of extant pathogens have identified many that have been associated with hominins since the Palaeolithic. Greenbaum *et al*. [39] have argued that an inter-species boundary between Neanderthals and modern humans in the Levant was maintained for tens of thousands of years during the Middle Palaeolithic by a disease barrier. Here we apply this reasoning to Upper Palaeolithic East Asia to explain why the southward spread of microblades stalled at the Qinling–Huaihe Line. Our disease hypothesis may afford a useful alternative to the bamboo hypothesis and various other proposals that are reviewed in Bar-Yosef *et al*. [12].

Peter *et al*. [116] investigated the role of geographical barriers in genetic differentiation, but the Qinling–Huaihe Line is not included in their list of such ‘troughs’. Importantly Bar-Yosef *et al*. [12] considered, but rejected, the ‘isolation hypothesis' for the absence of microlithic technology in southern China and Southeast Asia during the Palaeolithic. On the other hand, ancient DNA evidence for the north–south divergence of East Asian foragers (e.g. [54]) suggests that the Qinling–Huaihe Line may indeed have limited migration.

We have not mentioned palaeoclimate, except implicitly in connection with the initial distribution of malaria. Yi *et al*. [114] argue that, in the cold and harsh high-latitude environments where food was not easily obtained, microblades would have served as reliable, transportable and replaceable components of composite tools. They also claim that this technology spread from Siberia to northern China, among other northern regions, during the last glacial, but not to warmer southern China where resources were more homogeneously distributed. However, microliths are known from Middle Stone Age sub-Saharan Africa [2] and Upper Palaeolithic South Asia [6], so we would suggest that this may not be the exclusive, nor perhaps the primary, reason for the absence of microlithic industries in the south.

## Data Availability

The data are provided in the electronic supplementary material [117].
